# Spatial Separation and Working Memory Capacity Affect Selective Visual Attention in the Periphery

**DOI:** 10.3389/fpsyg.2021.692963

**Published:** 2021-09-16

**Authors:** Stefanie Klatt, Nicholas J. Smeeton

**Affiliations:** ^1^Institute of Sports Science, University of Rostock, Rostock, Germany; ^2^School of Sport and Health Sciences, University of Brighton, Eastbourne, United Kingdom; ^3^Institute of Exercise Training and Sport Informatics, German Sport University Cologne, Cologne, Germany

**Keywords:** allocation task, attention window, controlled attention, object recognition, visual field

## Abstract

The current study aimed to examine the effects of spatial separation and working memory capacity on selective visual attention. We investigated differences in the ability to identify the two covertly attended stimuli that appeared either along one of the meridians (e.g., both along the horizontal) or along two of the meridians (e.g., one along the horizontal and one along the vertical) in the attention-window task. Two visual stimuli in the periphery could be perceived along wider extents of the attentional focus’ meridians (horizontal, vertical, and diagonal) when they were located along the same meridian (e.g., horizontal) compared to two different ones (e.g., horizontal and vertical). Subjects with high working memory capacity outperformed subjects with lower working memory capacity in both conditions and stimuli presented on two meridians were less accurately perceived. The findings support the proposal that individual differences in working memory capacity are important for selective spatial visual attention.

## Introduction

Various researchers assume that differences in individuals’ working memory capacity are reflected in the distribution of their visual focus of attention (e.g., Bleckley et al., 2015). Working memory capacity is normally defined as the ability to hold unitized information in immediate awareness so that it can be manipulated and transformed into a more useful form (Schneider and McGrew, 2012). The visual focus of attention usually describes the area of the visual field in which subjects can be aware of and processes visual stimuli (Posner and Boies, 1971; Cowan, 1995). A number of studies have found that individual differences in working memory capacity can arise either from the ability to maintain information in the primary memory or the ability to manipulate the focus of attention to search for information stored in the long-term memory (Unsworth and Engle, 2007).

Two widespread tasks measuring the orientation of the focus of attention – the antisaccade (Hallett, 1978) and the flanker task (Eriksen and Eriksen, 1974) – confirm the relationship between attention and working memory capacity. While the anti-saccade task requires subjects to move their eyes and attention away from a strong visual cue, in the flanker task, participants are required to respond to the direction of a central arrow surrounded by congruent or incongruent arrows (flankers). Reaction times normally increase when the flankers are incompatible with the target. Lavie et al. showed that this flanker interference effect is greater when working memory load is high relative to when it is low. This indicates that working memory is essential for optimal selective attentional performance (cf. Lavie et al., 2004; Lavie, 2005).

Kane et al. (2001) examined the extent to which subjects were able to ignore a visual cue stimulus presented on one side of their visual field and instead focus their attention on the opposite side on which a stimulus had to be identified. The participants in that study were required to complete two tasks – the prosaccade- and the anti-saccade-task. The challenge of the prosaccade-task was to identify stimuli that appeared at the same spot the respective cue stimuli had been presented on before. In the anti-saccade-task, participants had to identify stimuli appearing at the side opposite to the cue stimulus. The results of this study showed no performance differences in the prosaccade-task between two groups with different working memory capacities. However, in the anti-saccade-task, the group of participants with lower working memory capacity performed the stimulus identification task substantially slower. They therefore probably experienced difficulties in suppressing involuntary/reflexive shifts of attention to the cue stimuli on the opposite side of the visual field. Accordingly, participants with higher working memory capacities not only responded more quickly, but they also achieved a greater number of correct responses in the task than participants with less working memory capacities (Kane et al., 2001; Unsworth and Spillers, 2010; Redick et al., 2012). According to Poole and Kane (2009), it is easier for participants with higher working memory capacities to perceive stimuli and disregard disruptive influences at the same time. Eventually, Conway et al. (2001) found out that working memory capacity is highly relevant to the ability to focus attention on specific information and that, in addition, it supports the ability to suppress irrelevant information. Working memory contributes to controlling perceptual attention (e.g., by holding templates for targets of perceptual selection) and action (e.g., by holding task sets to implement our current goals; Oberauer, 2019). Therefore, one can assume that the subjects who can apportion their attention better, have a better working memory.

In a study by Egly and Homa (1984), single alphabetical letters were presented along one of the three concentric rings around the fixation. At the beginning of each trial, the participants were informed about the likely eccentricity of the letters (close, medium, and distant), with no advance information given in a control condition. As the identification performance was better when the participants received information in comparison to the control condition, the authors concluded that attention can be directed to ring-like segments of visual space of various eccentricities. Building on these findings, Bleckley et al. (2003) performed another study, in which the participants were required to identify a central letter while simultaneously locating a displaced letter flashed somewhere on one of the three concentric rings. The authors assumed that the subjects with low working memory capacity distribute their attention according to the spotlight model by Posner (1980), while the subjects with high working memory capacity are able to focus their attention on non-contiguous areas and objects simultaneously.

**Figure 1:**
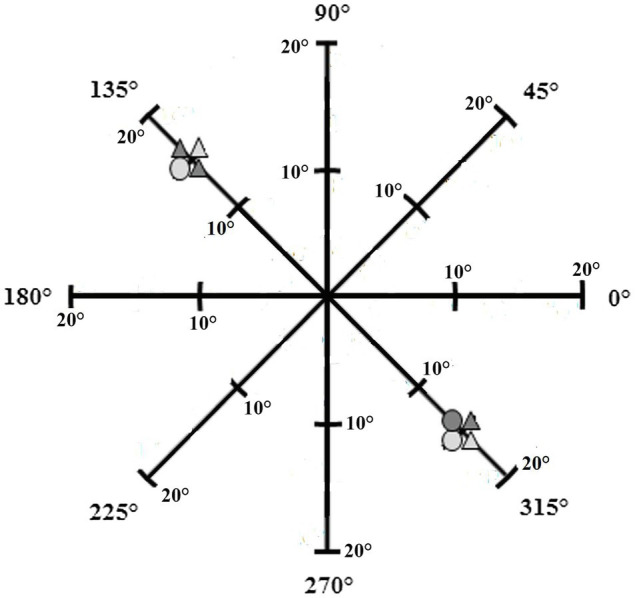
The stimuli were presented at eight distances from the center of the screen on four meridians (one horizontal, one vertical, and two diagonal) with eight directions (0°, 45°, 90°, 135°, 180°, 225°, 270°, and 315°) in the attention-window task. The figure represents a stimulus pair located on the diagonal meridian with a stimulus separation of 30° (from Hüttermann and Memmert, 2015).

Motivated by the approaches and findings of these previous studies, the relationship between individual working memory capacity and the distribution of spatial visual attention was investigated in the current study in more detail. While most research addressing the link between working memory capacity and spatial attention has required participants to fixate one target and process another one peripherally (e.g., selective attention task by Egly and Homa, 1984; see also Bleckley et al., 2003), the relationship has not yet been investigated in detail when the subjects are required to fixate two objects with equal priority in the visual periphery. There is only one study by Kreitz et al. (2015) that investigated the relationship between working memory capacity and the spatial visual attention when two peripheral objects had to be perceived simultaneously. The authors used the attention-window task developed by Hüttermann et al. (2013) as several studies have shown that this task is an adequate tool to determine attentional capabilities, requiring observers to attend to two equally attention demanding (peripheral) stimuli simultaneously (e.g., Hüttermann et al., 2019; Klatt and Smeeton, 2020; for an overview, see Hüttermann and Memmert, 2017). In the original task, these two peripheral stimuli are presented equidistant to the centre of a projection along one of the attentional focus’ meridians (one horizontal, one vertical, and two diagonal) with varying visual angles (separations) between the stimuli (see Figure 1). Kreitz et al. (2015) found correlations between working-memory measures and the accuracy rate when using the attention-window task. However, as usual in the original version of the attention-window task, the authors have only focused on situations in which both peripheral stimuli were presented along one meridian, e.g., along the horizontal or the vertical one.

Using a modified version of the attention-window task, the aim of the current study was to investigate if subjects with various working memory capacities allocate their attention differently when they are required to perceive two peripheral stimuli. For this purpose, the two peripheral stimuli were presented equidistant to the centre of the projection but, in contrast to the basic attention-window task, on different meridians, respectively (e.g., one stimulus on the horizontal and one stimulus on the vertical meridian). To determine participants’ working memory capacity, they were required to complete an automated version of the operation span task (Unsworth et al., 2005) in addition to the attention-window task in the current study.

Overall, based on the findings of previous research demonstrating a link between working memory capacity and flexibility of the allocation of attention (e.g., Bleckley et al., 2003), high working memory capacity individuals were expected to show better performances independent of the task condition compared to low capacity individuals. The stimuli in the basic and modified attention-window tasks were presented along eccentric circles around the fixation point (i.e., always with the same distance/eccentricity to the middle of the projection screen; e.g., with a visual angle of 10° on the vertical and horizontal meridians respectively). Based on this setup and on the findings of Bleckley et al. (2003), showing that low-working memory capacity participants normally allocate attention as a spotlight, it was hypothesized that low-working memory capacity participants would identify stimuli equidistant to the center of the projection equally well in both versions of the attention-window task. Furthermore, it was hypothesized that performances in high working memory capacity individuals would also be equally well in both task conditions as these individuals are able to flexibly allocate their visual attention. In addition, high working memory capacity individuals were expected to show better performances independent of the task condition compared to low capacity individuals.

## Materials and Methods

### Participants

Thirty-nine participants (14 females) aged 20–30years (*M*_age_=24.35years, SD=2.56years) took part in the study. Data from one participant was excluded due to low math accuracy (<85%) in the automated version of the operation span task (Aospan task; cf. Unsworth et al., 2005). All participants reported normal or corrected-to-normal vision (with contact lenses). Informed consent was obtained from each participant prior to testing according to the Declaration of Helsinki, and ethical approval was obtained from the lead institution.

### Materials and Procedure

Each participant performed the Aospan task as well as two versions of the attention-window task in a laboratory – one version in which both target stimuli were presented along the same meridian (original attention-window task cf. Hüttermann et al., 2014), and the other in which both stimuli were presented along two different meridians (e.g., along the horizontal and the vertical). Participants performed the three tasks (Aospan, basic attention-window, modified attention-window task) in random order. The completion of each task lasted about 10–15 min depending on the participants’ speed of responses.

#### Aospan Task

The Aospan task was programmed and run using E-Prime 2.0 (Psychology Software Tools, Pittsburgh, PA; cf. Unsworth et al., 2005). Participants carried out the task sitting at a distance of approximately 50cm in front of a 13-inch display (resolution: 1,366×768 pixels). Instructions were delivered on the screen prior to the task, and participants were encouraged to ask the experimenter questions prior to starting.

The Ospan score was used to differentiate participants into the two groups (low and high working memory capacity). Previously, researchers have demonstrated that the Aospan task is a reliable and valid indicator of working memory capacity (e.g., Redick et al., 2012). The task requires participants to decide on the correctness (true vs. false) of simple mathematical exercises (e.g., 13−7=5) while simultaneously trying to memorize a series of letters. Participants completed two short practice sessions, one for each of the subtasks (i.e., math exercise and remembering letters), before starting the main task. In each trial, participants first had to solve the math exercise before being presented for 1s with the letter they needed to memorize. Immediately afterwards, another math exercise was to be solved followed by a further letter, then another math exercise, and so on. After a set of three to seven operation-letter pairs, participants were required to recall all letters from the current set in the correct order by selecting the letters one after another from a display on the monitor. In total, the Aospan task included 15 trials (three trials each with 3, 4, 5, 6, and 7 letters to memorize). In line with the standard procedure concerning the data evaluation (cf. Unsworth et al., 2005), the Ospan score (i.e., the measure of a participant’s working memory capacity) was calculated as the sum of letters recalled across all error-free trials. Participants were informed about the necessity to keep their math accuracy at or above 85% at all times; during the recall of the letters, a percentage indicating the current accuracy was displayed in red in the upper right-hand corner of the screen.

#### Attention-Window Task (Basic Task)

**Figure 2:**
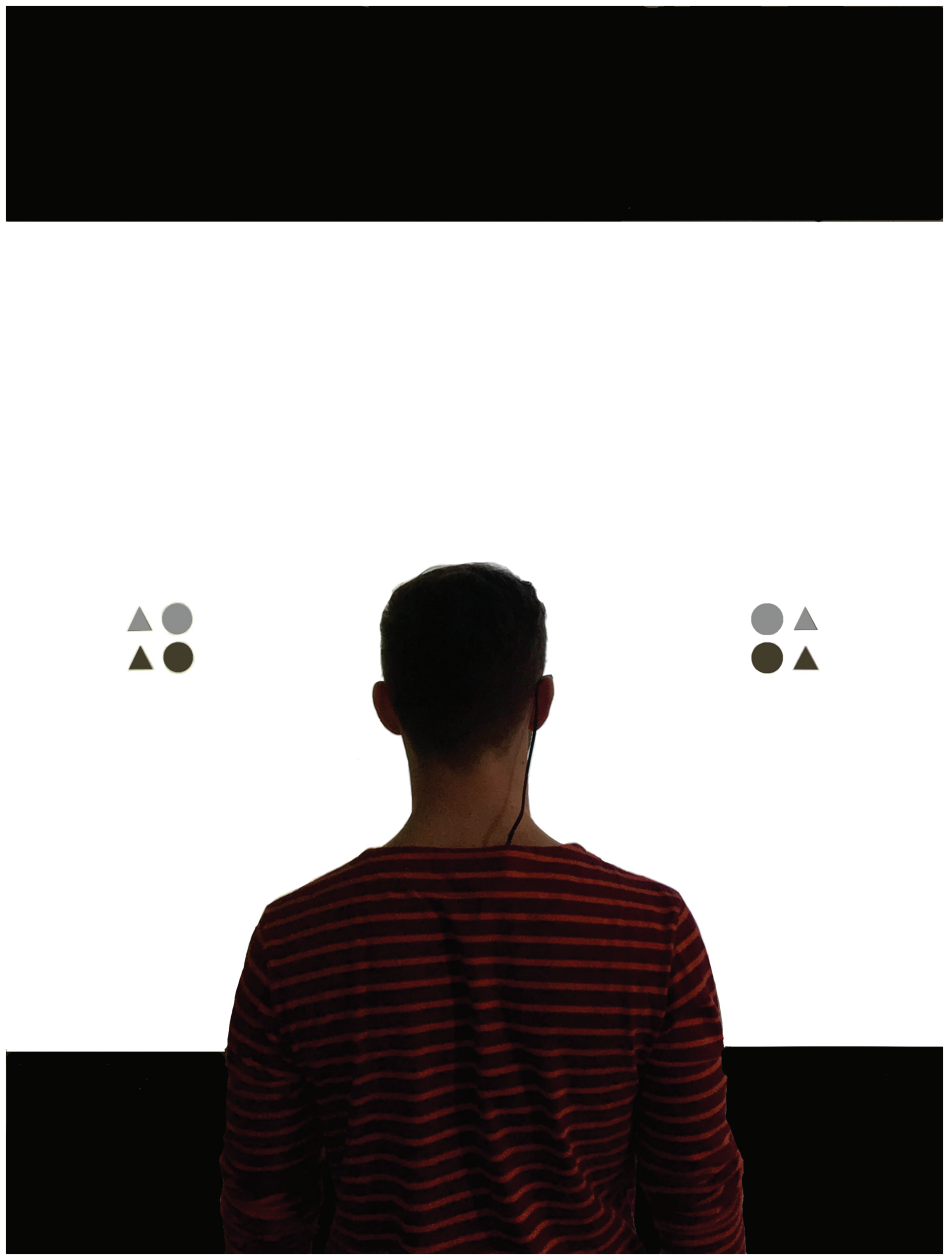
The experimental setup with a participant wearing the mobile eye-tracking system and standing in front of the screen while completing the attention window task with stimuli presented along the horizontal meridian.

**Figure 3:**
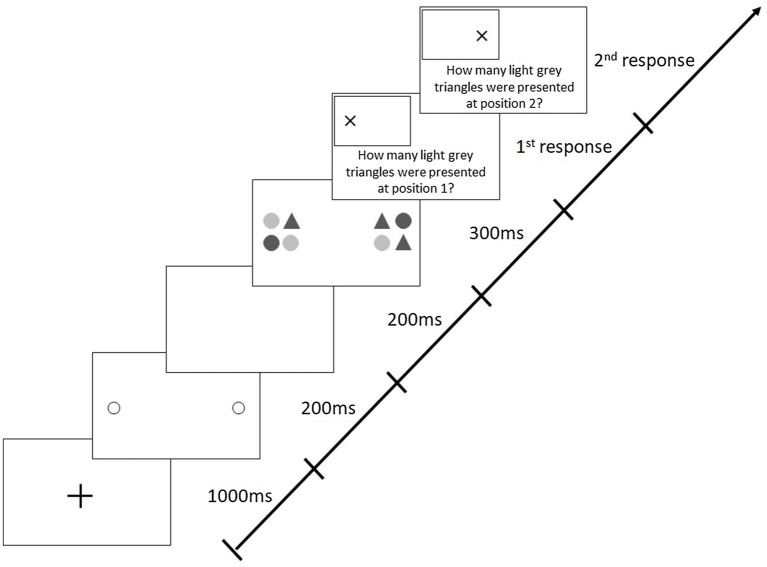
Sequence of events in a trial with stimuli along the horizontal meridian in the original attention-window task (adapted from Klatt and Memmert, 2021).

The attention-window task was programmed and run in E-Prime 2.0 (Psychology Software Tools, Pittsburgh, PA). For the implementation of the task, participants stood approximately 1.30m away from a 2.80m×2.20m screen (see Figure 2). Instructions for the attention-window task appeared on the screen, and participants were encouraged to ask questions before commencing the task. The participants’ task was to identify the number of light grey triangles presented in two peripheral stimuli. They responded verbally, and the experimenter entered the answers on a standard keyboard. After completing 16 practice trials, participants started the attention-window task, which consisted of three blocks of 112 trials each (i.e., 336 trials in total). Each trial began with a central fixation cross (1,000ms), followed by two pre-cue circles (200ms) indicating the future locations of the two peripheral stimuli. After a 200ms blank interval, the target stimuli appeared for 300ms (see Figure 3). Participants were required to fix their eyes on the central fixation cross throughout each trial in the attention-window task; fixation was monitored with a mobile video-based eye tracking system (SMI eye tracking glasses, 30Hz recording; SensoMotoric Instruments).

The different stimulus pairs displayed in the attention-window task were presented symmetrically around the centre of the screen, with distances between the stimuli ranging from 10° to 45° of visual angle (i.e., eccentricities of 5° to 22.5°) in increments of 5° of visual angle. The stimulus pairs were displayed along eight different meridional directions (0°, 45°, 90°, 135°, 180°, 225°, 270°, or 315°; in total one horizontal, one vertical, and two diagonal meridians). Stimuli positions varied randomly between all trials of each block. Each stimulus consisted of four elements. These elements were either circles or triangles (each corresponding to a size of 3.97°) coloured in light or dark grey, meaning there were four different possible elements in total. The shape (circle, triangle) and shade (light grey, dark grey) of all elements within each stimulus varied randomly between trials. There was an equal probability of 20% of the presentation of each zero, one, two, three, or four light grey triangles in each target stimulus. The participants’ task was to identify the number of light grey triangles presented in each stimulus. The task therefore not only required participants to detect simply the shape or the shade of the elements but rather the conjunction of both, that is, triangle and light grey, and was therefore attention demanding (Treisman and Gelade, 1980).

#### Attention-Window Task (Modified Version)

The modified version of the attention-window task was similar to the basic attention-window task except that stimuli were always presented along two different meridians (i.e., along the horizontal and the vertical, along the horizontal and one diagonal, or along the vertical and one diagonal meridian) in each trial. The combination of the two meridians stimuli were presented on was randomized and counterbalanced across all trials. However, the presentation of the stimuli was counterbalanced in this way that two objects could never been presented along two meridians lying next to each other, but there was always one meridian between both selected meridians.

### Data Analysis

By means of the median split method, participants were divided into two groups with either high working memory capacity (Ospan scores above the median split) or low working memory capacity (Ospan scores below the median split; for a similar procedure see, e.g., Sörqvist et al., 2012).

In the attention-window task, responses were only counted as correct if both stimuli were identified correctly. Based on the procedure of previous research using this task (e.g., Hüttermann et al., 2019), the attentional capability for peripheral vision was determined. It was examined how effectively participants adapt and align their visual attention, that is, how effectively they were able to correctly judge two peripheral stimuli along the attentional focus’ separate meridians. A mixed design analysis of variance (ANOVA) was conducted with accuracy rate as the dependent variable and task condition (one meridian presentation, two meridians presentation) as the within-participant factor and working memory level as between-participant factor.

## Results

In the Aospan task, participants achieved an average score of 58.46 (SD=13.57) out of a possible total value of 75. Using the median split method on the Ospan scores (median score: 62), half of the participants (*n*=20) were classified as high working memory individuals (*M*=68.50, SD=4.42) and the other half (*n*=19) as lower working memory individuals (*M*=47.89, SD=11.77). The Ospan scores significantly differed between participants with high working memory capacity and those with lower capacity, *t*(37)=7.310, *p*<0.001, *d*=2.342.

In the attention-window task, participants correctly identified 57.77% (SD=13.76%) of the stimuli across both task conditions. A visual inspection of the data confirmed that the participants had kept their eyes fixated on the center of the screen (see Brocher et al., 2018; Klatt et al., 2021, for a similar procedure). Therefore, none of the trials had to be excluded from the data analysis due to deviations from the required gaze behavior. The ANOVA revealed a main effect of condition, *F*(1, 37)=30.148, *p*<0.001, *η*^2^=0.449: Participants identified more stimuli correctly when stimuli were presented along one meridian (*M*=61.50%, SD=14.53%) compared to two different meridians (*M*=54.05%, SD=14.23%). Across both task conditions, participants with a higher working memory capacity (*M*=64.23%, SD=13.03%) were more accurate than those with less capacity (*M*=50.97%, SD=11.20%), *F*(1, 37)=11.561, *p*=0.002, *η*^2^=0.238. There was no significant interaction between working memory group and task condition, *F*(1, 37)=0.073, *p*=0.789.

## Discussion

The present study was guided by the assumption of Bleckley et al. (2003) that the visual attention of low working memory capacity individuals is distributed around the center of the focus of attention in eccentric circles whereas high working memory capacity individuals can adjust their attentional capabilities more flexibly. Thirty-nine participants performed two versions of an attention-demanding task, one in which two peripheral stimuli were presented equidistant to the center of the projection screen along one meridian of the attentional focus (e.g., vertical) and one in which these stimuli were presented along different meridians (e.g., vertical and horizontal) but also at the same distance to the projection center. The results showed that participants with high working memory capacities achieved better attentional performances in both versions of the attention-window task compared to participants with lower working memory capacities. This finding confirms previous research pointing out a link between working memory capacity and the allocation of visual attention. Previous studies that have highlighted that working memory capacity reflects the ability to control attention and constrain attention to relevant information (e.g., Kane and Engle, 2003; Redick and Engle, 2006; Heitz and Engle, 2007) could be supported and supplemented for situations in which two stimuli have to be identified simultaneously in the visual periphery.

Overall, in the current study, both groups – low and high working memory capacity individuals – performed better when both target stimuli were presented on the same meridian as opposed to two different ones. This pattern of results was observed even though the two respective target stimuli were always presented at the same distance to the center in both versions of the attention-window task (i.e., the eccentricities were the same in both task conditions) and the participants’ fixation position in the middle of the screen was controlled with a mobile eye-tracking system. This finding contradicts the assumption that subjects (at least with low working memory capacity) distribute their attention according to the spotlight model in the attention-window task. This assumption was made based on the study of Bleckley et al. (2003). The authors showed that the subjects with low working memory capacity allocate attention as a spotlight. However, they used a task requiring subjects to fixate on one central cue while simultaneously perceiving another cue in the periphery. Obviously, there are different mechanisms being responsible for the subjects’ spatial distribution of attention in both described tasks.

The most interesting and important finding of the current study is that participants performed worse in the two-meridian condition compared to the one-meridian condition. When two peripheral target stimuli have to be identified, it seems to be easier for participants if they only have to allocate their visual attention along one meridian compared to two meridians independent of the individuals’ working memory capacity. One possible explanation by Kootstra et al. (2008) for why target stimuli displayed on the same meridian may be perceived more accurately is that symmetrical constellations can be identified more easily than asymmetrical constellations. There is evidence that humans are highly sensitive to symmetrical patterns and pay attention to symmetry (Kaufman and Richards, 1969). With regard to the two task conditions, the participants performed on symmetrical patterns in the task with stimuli presented along the same meridian and asymmetrical patterns in the modified attention-window task. Perhaps, this is the reason that attention may be allocated more effectively with an elongation across the meridian rather than a division of attention across the visual field. However, presentation of more stimuli along the meridian line could address this question experimentally (Carrasco et al., 2001; Talgar and Carrasco, 2002).

There are some limitations and considerations for future research that need to be acknowledged. Although we found that individual differences in working-memory capacity vary with the ability to perceive two spatial objects in the periphery along either one or two meridians of a person’s visual attentional focus, we cannot infer from this correlation that changes in one capacity cause changes in the other. An experimental manipulation of one capacity is needed to check for any causal inference of one capacity affecting the other in future.

A further possible limitation of the current study is that, although the results provide insights into the link between working memory capacity and the allocation of visual attention, no reliable statements can be made about the entire area of the attentional focus of low and high working memory capacity individuals. It might be possible that these two groups differ in the orientation of their attention: For example, some subjects (high or low capacities) may be able to only focus on one of the different meridians of their visual focus of attention, while others may be able to focus on whole surfaces within their attentional focus. However, the fact that subjects seem to be able to perceive visual stimuli along different meridians simultaneously contradicts the assumption that attentional capabilities are only spread over one meridian and rather indicates a distribution along either at least two meridians or a larger surface. Furthermore, although the current study controlled for the position of participants’ visual fixations with an eye tracking system, the study design does not enable conclusions to be drawn about whether participants attended to both stimuli simultaneously or shifted attention rapidly from one stimulus to the other (e.g., Bichot et al., 1999; Scharlau, 2004). Future studies comparing different gaze strategies dependent on the subjects’ task performance are required. In these studies, one condition should examine whether subjects broaden their focus of attention in a way that these stimuli are all encompassed within one unitary focus, following the approach of the zoom lens model (cf. Eriksen and St. James, 1986). Another condition should investigate if the subjects have divided their attention between the two target stimuli (cf. Jefferies et al., 2014). Potentially, the participants with a high working memory capacity may have perceived the stimuli sequentially and then performed the analysis in the working memory rather than with the visible displays. If this is true, their performance advantage might result from more efficient attention shifts, coupled with superior working memory capacity (Bundesen, 1990).

A supplementary approach for future studies might be a modification in the calculation of the subjects’ attention window. So far, the subjects’ responses in each trial were only considered right if they were able to report the number of light gray triangles at both locations correctly. It was not analyzed to which direction an error was made (e.g., if a participant failed to report the number of light gray triangles on the left side, but was able to name the correct number on the right side, the overall answer was considered wrong). If this calculation mode undergoes a change in future studies, it would make it possible for the subjects to attended to both stimuli simultaneously or shift attention rapidly from one stimulus to the other.

Another possible limitation of the current study is that in the modified attention-window task, in which stimuli were presented on different meridians, performances for the combinations of meridians were not considered separately from each other. This means that the performance was averaged across all trials, even though it might be possible that the worse performance in the modified version compared to the basic attention-window task could be attributed to only single combinations (e.g., combination of horizontal and diagonal meridian) and not to all combinations (e.g., not to horizontal and vertical). Future studies should systematically analyze these differences in more detail.

In the current study, the two target stimuli always appeared equidistant from the center of the screen. Future research should explore differences in attention allocation by varying target positions independent of each other along the different meridians. A further approach for future studies with the attention-window task might be to use invalid cue stimuli (such as in the antisaccade-task by Kane et al., 2001) in order to explore whether subjects are able to ignore these cues and especially to examine possible differences between high and lower working memory capacity individuals. Based on the findings of Kane et al. (2001) it should be expected that individuals with low working memory capacities would have more difficulties suppressing invalid cues which would probably lead to a smaller number of correct responses in such a modified version of the attention-window task.

In sum, a new version of the attention-window task was introduced demonstrating that, independent of working memory capacity, it is more difficult for individuals to perceive two peripheral stimuli, at the same distance to the fixation position, along two different meridians compared to conditions in which stimuli are presented along the same meridian. Furthermore, the current study confirmed that high working memory capacity individuals outperform low working memory capacity individuals in perceiving visual targets in the periphery independent of the task condition.

## Data Availability Statement

The raw data supporting the conclusions of this article will be made available from the authors on request.

## Ethics Statement

The study was conducted according to the guidelines of the Declaration of Helsinki and approved by the ethics committee of the German Sport University Cologne. Informed consent was obtained from all subjects involved in the study.

## Author Contributions

SK developed the study concept, collected the data, and wrote the first draft of the manuscript. Both authors contributed to the article and approved the submitted version.

## Conflict of Interest

The authors acknowledge financial support by the German Research Foundation and the University of Rostock within the funding program Open Access Publishing.

## Publisher’s Note

All claims expressed in this article are solely those of the authors and do not necessarily represent those of their affiliated organizations, or those of the publisher, the editors and the reviewers. Any product that may be evaluated in this article, or claim that may be made by its manufacturer, is not guaranteed or endorsed by the publisher.
